# Infant feeding modalities addressed in two different ways in Eastern Uganda

**DOI:** 10.1186/1746-4358-5-2

**Published:** 2010-02-04

**Authors:** Ingunn MS Engebretsen, Rebecca Shanmugam, A Elisabeth Sommerfelt, James K Tumwine, Thorkild Tylleskär

**Affiliations:** 1 University of Bergen, Centre for International Health, Bergen, Norway; 2 Medical Research Council, Biostatistics Unit, Ridge Road, Overport, South Africa; 3 Academy for Educational Development, Washington, DC, USA; 4 Makerere University, Department of Paediatrics and Child Health, Kampala, Uganda

## Abstract

**Background:**

Durations of exclusive breastfeeding (EBF) and predominant breastfeeding (PBF) from two different assessments, among the same mother-infant population, were investigated to determine the degree to which the assessments yielded overlapping results.

**Methods:**

Thirty Ugandan mother-infant pairs were followed up weekly from birth to three months of age with weekly short-time feeding recall: the 24-hour recall asked prior to the 1-week recall. In addition, at week 6 and 12 dietary recalls since-birth were conducted. Variables for the duration of EBF and PBF were created from the short-time feeding recalls and the dietary recalls since-birth, respectively. Mean durations of EBF and PBF from the two assessments were compared with Kaplan Meier analysis at week 6 and 12. Reproducibility of dietary recall instruments was also assessed.

**Results:**

At six weeks postpartum the mean durations of EBF were 0.50 weeks (95% CI: 0, 1.02) according to the weekly short-time recalls and 1.51 weeks (95% CI: 0.66, 2.35) according to the recall since-birth (Mantel-Cox test, p = 0.049). The mean durations of PBF were 4.07 weeks (95% CI: 3.38, 4.77) according to the frequent short-time recalls and 4.50 weeks (95% CI: 3.93, 5.07) according to the recall since-birth, (Mantel-Cox-test, p = 0.82). At twelve weeks the mean durations of EBF were 0.5 weeks (95% CI: 0, 1.1) according to the weekly short-time recalls and 1.4 weeks (95% CI: 0.1, 2.7) according to the recall since-birth (Mantel-Cox-test, p = 0.15). The mean durations of PBF were 5.2 weeks (95% CI: 3.9, 6.5) according to the weekly short-time recalls and 6.6 weeks (95% CI: 5.4, 7.8) according to recall since-birth (Mantel-Cox-test, p = 0.20). Reports of feeding categories and early feeding practices showed high reproducibility.

**Conclusion:**

Comparing duration of EBF and PBF in this group of mother-infant pairs showed overlapping results from the weekly short-time assessment and the recall since-birth at twelve weeks, with the latter yielding slightly longer duration of the respective feeding modalities. The retrospective recall since-birth could be assessed as a cost-reducing tool compared to the frequent follow-up addressing duration of respective infant feeding modalities for evaluation of programmes promoting safer infant feeding practices.

**Trial registration:**

The study was part of formative studies for the ongoing study PROMISE EBF registered at http://clinicaltrials.gov, NCT00397150.

## Background

Breastfeeding has the potential to save neonatal, infant and young child lives and to reduce morbidity [1]. It is estimated that promotion of exclusive breastfeeding (EBF) for six months, roughly defined as giving nothing but breast milk except vitamins, minerals or medicines, could prevent 8% of global annual child mortality [2]. Breastfeeding is ranked as one of the safest and most efficient health interventions to achieve the millennium development goal 4 (MDG4): reduce child mortality [3,4]. After field studies clearly had shown how HIV could be transmitted through breast milk [5], infant feeding practices received close attention [6]. Today there is agreement that EBF is just as safe as or even safer than replacement feeding when it comes to HIV-free survival in resource-deprived settings [7]. Newly released studies from 2009 have seen very low post-natal transmission rates when either 1) the mother is on multi-drug anti-retroviral treatment or 2) HIV-negative children of HIV-positive mothers are on peri- and post exposure prophylaxis during lactation [8]. This resulted in recent changes in the guidelines. The World Health Organization's (WHO) recommendations on HIV and infant feeding from 2006 said [9]: exclusive breastfeeding is now recommended for the first 6 months of life unless replacement feeding is acceptable, feasible, affordable, sustainable and safe for the mothers and infants before that time. The recommendations and research might seem clear. However, the recommendations are difficult to follow for most HIV-positive mothers in resource-poor settings [10], because of the stigma and limited feasibility that results in negligible numbers of both exclusively breastfed and exclusively replacement fed infants in the most highly HIV-burdened areas [11,12]. Even if new treatment regimens with breastfeeding will be recommended at an even higher degree in the years to come for HIV-positive mothers in resource poor settings [13], challenges may remain for mothers regarding feeding issues. Infant feeding will remain one of the most important aspects of reduced postnatal mother-to-child transmission as a disproportionately low percentage of pregnant women (12%) have access to the necessary treatment and care for their own health and only 33% in medium- and low income countries have access to drug regimens to avoid mother-to-child transmission with the simple drug regimens existing today [14]. The prevalence of EBF is also low in the general population; both because of sub-optimal promotion of EBF and a spill-over effect in the HIV-negative or HIV-unknown population from the HIV preventive work with an increased tendency towards mixed feeding (MF) after birth (see definition below) [15,16]. EBF promotion needs renewed emphasis in the general population and renewed efforts will benefit all children.

Breastfeeding prevalence has usually increased with lay breastfeeding promotion, but varying results have been seen for EBF rates [17]. A recent review identified pitfalls and future challenges in the rolling-out of exclusive breastfeeding and improved complementary feeding promotion [18]. Monitoring of breastfeeding and EBF practices have also varied with different studies [19]. WHO's definitions are as follows: (1) Exclusive breastfeeding (EBF): the infant has received only breast milk from his/her mother or a wet nurse, or expressed breast milk, and no other liquids, or solids with the exception of drops or syrups consisting of vitamins, mineral supplements or medicines [20] (2) Predominant breastfeeding (PBF): the infant's predominant source of nourishment has been breast milk. However, the infant may also have received water or water-based drinks (sweetened or flavoured water, teas, infusions, etc.); fruit juice; Oral Rehydration Salts (ORS); drop and syrup forms of vitamins, minerals, and medicines; and folk fluids (in limited quantities). With the exception of fruit juice and sugar-water, no food-based fluid is allowed under this definition [20]. (3) Mixed feeding (MF): feeding both breast milk and other foods or liquids [15]. MF has the same meaning as 'partial breastfeeding' - giving a baby some breast milk, and some artificial feeds, either milk or cereal, or other food, but MF is more frequently used in the context of postnatal mother-to-child transmission. Both MF and 'partial breastfeeding' are used when the quality aspects of the food is disregarded. Complementary feeding comprises of a certain level of food quality in its recommendations and is defined as foods given after six months. EBF, PBF and MF are the terms used in this paper. One conservative way to interpret these definitions is to emphasize the words 'has received' in such a way that if a single item belonging to category two or three above has been given to the infant, that infant belongs to category two or three, respectively. As a consequence of this way of interpreting the definitions, the infant cannot be re-defined into a prior category: the duration of EBF will then be defined as up to the start of PBF, and the duration of PBF as up to the start of MF, on the premise that PBF is introduced prior to MF. An alternative to the conservative interpretation mentioned above is the 'current status' description highlighting what the prevalence of certain behaviours are 'now' (often based on a 24-hour, 48-hour or 1-week recall) [21]. This paper will utilise the conservative interpretation of the definitions and not the current status definitions.

In the context of HIV and infant feeding WHO launched a tool for research in 2001 to capture infant feeding practices [20]. In that tool frequent (preferably weekly) 7-day infant feeding recalls was recommended in order to get continuous assessment. The 7-day infant feeding assessment was recommended based on a South-African validation study, among other studies [22]. The design with weekly 7-day recalls has been utilised in a few settings, including a non-randomised intervention trial assessing HIV-transmission and infant feeding practices [7]. However, even if the tool from 2001 provides comprehensive questionnaire instruments, almost a gold standard of prospective population-based infant feeding assessment, the authors of the tool emphasize that not all kinds of studies should use it. That is because the cost of such an assessment is overwhelming for most low-resource studies and for programme evaluation purposes. On the other hand, the 24-hour recall approach is widely used and often the basis for categorising infants into EBF, PBF, MF and replacement fed (RF) [23]. By capturing the 'current' practice in infant populations, only proportions practicing respective feeding modalities can be calculated within different age groups [21]. It would be useful to have a tool which could provide the duration of feeding modalities in addition to current status information without using the prospective weekly 7-day infant feeding recalls that are too costly and labour intensive for most study purposes.

In this study, in a resource poor setting in Eastern Uganda, estimates of the duration of EBF and PBF were compared in the same group of mother-infant pairs by two different methods. The comparisons were based on (1) weekly follow-up of mother-infant pairs where a 24-hour dietary recall was asked prior to a 1-week infant feeding recall, this was a modified version of the tools proposed by WHO [20]; and (2) recall since-birth conducted at week six and again at week twelve. Reported information on colostrum, pre-lacteal feeding and initiation of breastfeeding were assessed at week 1 and again at week 3 after birth and consistency was assessed. In addition, the reliability of variables created for different feeding modalities was assessed.

## Methods

### Site and study population

Mother-infant pairs for this comparison study were recruited in Mbale District, Eastern Uganda, as part of formative studies for the larger study, PROMISE EBF: Safety and Efficacy of Exclusive Breastfeeding Promotion in the Era of HIV in Sub-Saharan Africa (Id: NCT00397150 at http://clinicaltrials.gov). Data collection is finished for the PROMISE EBF study which was a multi-site cluster-randomised behavioural-intervention study across four African countries [24]. Mbale Municipality is a town of approximately 75,000 inhabitants, 200 kilometres from Kampala. The population is mainly Bagisu and some areas are influenced by migration, mainly due to unrest further north, resulting in the development of semi-permanent housing areas and language challenges. The population mainly comprises of subsistence farmers, not only in the rural areas but also to a large extent in the urban areas. There was no need for external translators among the mothers participating in this study.

Mothers who had delivered within seven days of first contact were the primary target as respondents. Half-a-year prior to the start-up of PROMISE EBF data collection and intervention, from 13 June to 27 June 2005, mothers were consecutively recruited for this comparison study from clusters within the PROMISE EBF study setup. They all resided within a maximum driving distance of one hour from the centre of Mbale Municipality. The study team approached them through recruiters residing within the respective clusters equivalent to one or two villages. Each recruiter reported to the study coordinators every birth and pregnant woman with gestational age greater than seven months within their cluster. Thirty-one mothers were approached, and thirty consented to participate in this comparison study. Three were lost to follow up, of whom one baby died (Table 1). There was no evidence of family relations or strong social bonding between any of the thirty mothers. The mother-infant pairs were followed up weekly for 12 weeks. A mother was considered lost to follow-up if there were three or more consecutive missing weekly interviews or other reasons for non-participation were given. All together 427 interviews were performed (Table 1).

**Table 1:** Study overview

Interview type and time	**Recall types**^**1**^	Planned interviews	Performed interviews	Reproducibility check	Missing interviews	**LTFU**^**2**^	**Reason LTFU**^**2**^
Recruitment	Baseline	30	30	27			
1st week	EIFP, ^3 ^24-h, 1-wk,	30	29		1		
2nd week	24-h, 1-wk	30	29		1		
3rd week	EIFP,^3 ^24-h, 1-wk, ever	30	30		0		
4th week	24-h, 1-wk	30	28		1	1	Moved
5th week	24-h, 1-wk	30	28			1	Moved
6th week	24-h, 1-wk, ever	30	28	27			
7th week	24-h, 1-wk	30	26		2		
8th week	24-h, 1-wk	30	24		4		
9th week	24-h, 1-wk	30	27		1		
10th week	24-h, 1-wk	30	25		3		
11th week	24-h, 1-wk	30	26		1	1	Death^4^
12th week	24-h, 1-wk, ever	30	25	18	2		
Total		390	355	72	16	3	

Planned, performed and extra interviews conducted for 'reproducibility check' at different time points. Recall types, missing interviews and loss to-follow-up with reasons given.

35 (9%) missed interviews

3 (10%) losses-to-follow up during 12 weeks

^1^The recall types are listed in the order they were asked: 24-hour recall (24-h), 1-week recall (1 wk) and recall since-birth (ever).

^2^LTFU = Loss-to-follow up

^3^EIFP = Early infant-feeding practices

^4^Infant death

### Questionnaire, design and data collection

The structured interviews were translated and back-translated from English to Lumasaaba. Five data collectors who were fluent in the local language, Lumasaaba, conducted the interviews. The mothers were asked about breastfeeding practices from when 'she woke up yesterday morning till this morning,' and 'last week' at each weekly visit. In addition, mothers were asked questions from the 22-item dietary recall lists, containing foods and liquids site-specifically chosen and pre-tested, in a 24-hour and 1-week recall at each weekly visit. This was in line with the approach from the WHO 2001 tool [20]. In the 24-hour dietary recall mothers were asked: 'From the time you woke up yesterday morning till you woke up this morning: did you give any of the following items to the child?' In the 1-week recall the mothers were asked: 'Now I am going to ask you if you gave the following items at all the last week, etc.' These two questions combined are referred to as 'frequent short-time recall' in this paper. At week 6 and 12 only, the mothers were asked: 'Now I am going to ask you if you ever have given the following to your baby and if you have done that, please tell us when you did that for the first time.' In this paper the last approach is referred to as 'since-birth recall.' The items listed were the basis for categorising the feeding practices into EBF (breast milk, syrups and medicines only), PBF (in addition to breast milk: water, water with sugar or glucose, fruit juice, tea without milk and gripe water) and MF (breast milk in addition to tea with milk, rice water (thick), diluted cow's milk, undiluted cow's milk, infant formula, powdered milk other than infant formula, any dairy product such as yoghurt, cheese or cream, goat milk, cereals, porridge/bread, fruits or vegetables, meat, fish and egg). Foods and liquids allowed in the PBF category were allowed in the MF category, but not vice versa. Other items were probed for. All mothers breastfed throughout the study, so there was no need to classify any as RF.

In the weekly longitudinal assessment, having given an item once or more in the PBF or MF categories qualified for changing the respective infant's feeding modality that first week it was introduced, irrespective of whether the mother reported the item in the 24-hour or the 1-week dietary recall. If a mother was absent from weekly interviews and the infant changed feeding category between one interview and the next, it was estimated that the infant changed feeding mode midway between the two interviews. If the feeding mode did not change between the two interviews it was interpreted as no change in the feeding pattern.

### Data analysis and definitions

Time of introduction of feeds qualifying for PBF and MF according to the since-birth recall was compared to the time of introduction of feeds according to the frequent short-time recall and analysed at week 6 and week 12 after birth. Kaplan-Meier analysis was used to compare the two approaches, and a Mantel-Cox test was used to assess whether they were equivalent.

Information from the interviews conducted twice at week 6 and 12 were used to assess the reproducibility of some central study questions, including the derived feeding categories. The two interviews conducted at different points in time at week 6 and 12, respectively, utilised identical questionnaires: one was allocated the role of 'the standard interview' and the other of 'the reproducibility interview.' Whether the reproducibility interview was conducted first or second was randomly selected according to a random number list. One interview was performed in the morning and the other in the afternoon, with a minimum of five hours between them. Different data collectors performed the interviews in the morning and the afternoon, so one mother was not seen by the same person twice in the same day. The aim was to cover the same period in the 24-hour and 1-week dietary recall, while minimising the mother's memory of the previous answers. Twenty-seven mothers volunteered for two interviews at week 6 and eighteen at week 12 for the purpose of reproducibility checking. Reproducibility of 'early feeding practices' was assessed by comparing answers from week 1 and 3.

The assumption was that infants could be categorised into EBF, PBF and MF consecutively. This implies that EBF has ended and PBF started when water-based solutions and fruit juices are introduced, and that PBF has ended and MF started when milk-based solutions and semi-solid feeds are introduced. This assumption might not always be true: for example, an infant can be given milk-based solutions without having been given water first, which makes that particular infant skip the predominant breastfeeding category. Calculating the introduction of PBF is not meaningful if items qualifying for MF have already been introduced. Therefore, the initiation of PBF was controlled for and adjusted according to the introduction of MF, if that was introduced prior to PBF. In the frequent short-time recalls, PBF was introduced prior to MF among all the mothers, but according to the recall since-birth, three cases in the six week interview and two cases in the twelve week interview answered the other way round.

There has been differing interpretation about the approach emphasizing 'has received' in the WHO definitions. Infants can go 'in' and 'out' of feeding categories. They can be given items qualifying for the predominant breastfeeding or mixed feeding categories for a period of time and thereafter be exclusively breastfed again [20]. Breastfeeding can also be interrupted and started again. Illustrations were made from the weekly recalls where one cell represented one week for one mother-infant pair and items qualifying for PBF and MF, respectively, were given different patterns. Missed visits were also marked.

Sensitivity and specificity for rates of EBF and MF at six and twelve weeks were calculated. Sensitivity was defined as the proportions estimated to be 'EBF' and 'MF' in the recall since-birth at week 6 and 12 among those who were estimated to be 'truly EBF' and 'truly MF' in the frequent short-time recalls. Specificity was defined as the proportions estimated to be 'not EBF' or 'not MF' in recall since-birth at week 6 and 12 among those who were estimated to be 'truly not EBF' and 'truly not MF' in the frequent short-time recalls.

Pre-lacteal feeding was assessed in the questionnaire the following way: "Within the first three days after birth was the baby given anything to drink?" As most of the babies already were put on the breast within the 3-day period the term 'pre-lacteal' is somehow misleading. It could also be called 'feeds prior to lactation establishment,' or 'first 3- days feeds,' etc. We have chosen to keep the term 'pre-lacteal feeds' for its conventional use.

Answers about pre-lacteal feeding and early feeding practices which were assessed in the 1 week interview and 3 week interview were compared. Cohen's kappa (κ) was calculated and reported. An additional check was added in the form of a one-sample test of proportion. It is well known that there is wide disagreement about the use of Cohen's kappa to assess inter-rater agreement [25]. There are important sources which give compelling arguments and discuss the pros and cons of Cohen's kappa [26]. In view of this critique, it was decided to use the simple proportion test in addition to the kappa test, since it is still widely used and accepted as a measure of inter-rater agreement.

For the proportion test, the proportion selected *a priori *as the 'gold standard' to judge agreement was 70%. Thus, if agreement was seen to be statistically less than 70%, the proportion test would imply that the gold standard was not met, so the comparison was discordant (these comparisons were made at the 5% level of significance). The 70% level was selected since it was deemed to be a strict cut-off, i.e. at least 21 of the 30 mothers must give concordant answers. Concordance was also tested with respect to the reproducibility of the variables created for PBF and MF at weeks 6 and 12. The level of agreement was also set to 70%.

Data were double entered using EpiData 3.0 [27] and analysed using SPSS 15.0.1 and STATA/IC 10.1. Descriptive statistics were calculated. Mean and median were used for continuous data and Fisher's exact test for categorical data.

### Ethics

All participants signed/gave thumb print to informed consent forms. The mothers were told there were no direct benefits for participating in the study and that participation did not influence any other health services. They were informed about the planned frequency of visits and how time-demanding they would be. Interview times ranged from 15 to 45 minutes. The data collector referred the mother and/or household members to the nearest health centre in the event of any obvious illness in the household. Ethical approval was obtained from Makerere University Faculty of Medicine Ethics and Research Committee.

## Results

Of the 30 mothers, one did not know her age. The remaining 29 were from 15 to 36 years old; median age was 24 years. Three mothers had never attended school and the rest had attended school for between 3 and 11 years; the median school attendance was 7 years. Nearly half (13/30) reported that they were unable to read and write. The high level of illiteracy reflected varying quality of the education depending on teachers, time, site, school material, size of classes, etc. Education had often been interrupted due to lack of school money, and there were low reading and writing stimulation for women after primary education. Four out of 30 had never attended an antenatal care unit (ANC). The median number of visits among the 26 mothers who had visited ANC was 3. Half of the mothers had been informed about HIV voluntary counselling and testing (VCT), twelve had received the service, and 10 had been tested. Six knew their HIV results and four did not. Twenty-three mothers had previously had babies and had breastfed their infants. All, but one, provided information about site of birth: 27 (93%) delivered at home, unassisted, 1 (3%) delivered at home with the assistance from a friends and/or family, and 1 (3%) delivered in the traditional birth attendant's place. None of 29 mothers provided information about assistance from health workers. Socio-economic baseline characteristics are given in Table 2. The population can be described as relatively poor: few mothers had items ranked as expensive (cupboard, telephone) and none had a refrigerator or gas/electric heater. All the mothers used wood or charcoal as their fuel for cooking. Half of them had access to public tap water; only one had water piped into her yard. Five had access to electricity, while 24 had electricity in their village. House walls and floors were mainly of earth/dung and roofs mainly of iron sheets for 90% of the mothers.

**Table 2:** Baseline characteristics

Socio-economic indices	n	%
Urban/rural status		
Mbale Municipality (urban)	19	63
Bungokho (rural)	11	37
Mother's age		
24 years and less	15	50
25 years and above	14	47
Education		
Not attended	3	10
3-6 years in primary	13	43
Finished P7 and above	14	47
Marital status		
Married/cohabiting	23	77
Single	7	23
Parity		
1(index infant)	7	23
2 to 3	8	27
4 and above	15	50
Items in working condition		
Radio	14	47
Lantern	14	47
Bicycle	7	23
Phone/mobile phone	5	17
Cupboard	3	10
Animals	4	13
Motor cycle/scooter/truck	2	7
Number of pair of shoes		
1-2	25	83
3-4	5	17
Number of foam matresses		
0-1	13	43
2	13	43
3-5	4	13

Baseline characteristics and socio-economic assets among 30 Eastern Ugandan mothers

### Comparisons of recall assessments

**Figure 1:**
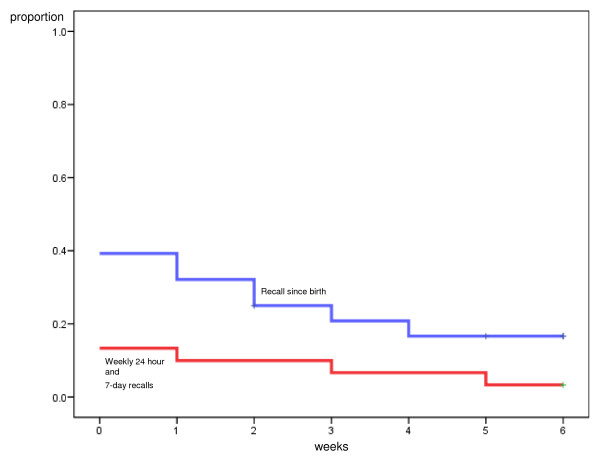
**Comparison of short-time and 'since birth' dietary assessments for introduction of predominant breastfeeding (PBF), 6 weeks**. Kaplan-Meier curves illustrating proportion introducing water, water-based drinks, oral rehydration salts or fruit juices to breastfed infants (PBF) according to the frequent short-time recall, 24-hour recall and weekly 1-week recall (red) and recall 'since-birth' (blue) dietary assessments at six weeks.

**Figure 2:**
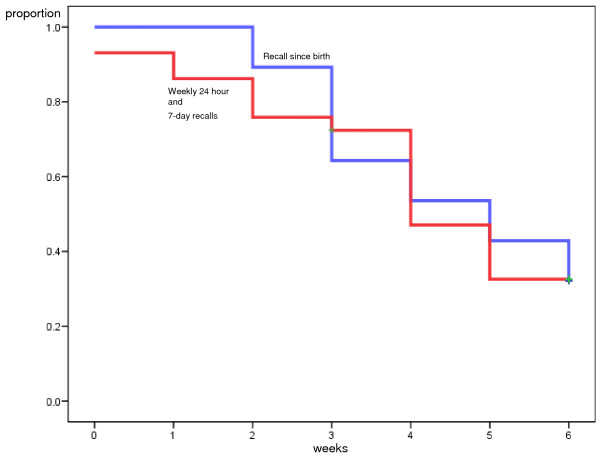
**Comparison of short-time and 'since birth' dietary assessments for introduction of mixed feeding (MF), 6 weeks**. Kaplan-Meier curves illustrating proportion introducing milk and semi-solid feeds to breastfed infants (MF) according to the frequent short-time recall, 24-hour recall and weekly 1-week recall (red) and recall 'since-birth' (blue) dietary assessments at six weeks.

The following mean duration of EBF (start of PBF) and PBF (start of MF) were seen at week 6 and 12 according to the Kaplan-Meier analysis when comparing the frequent short-time recall with the recall since-birth: At six weeks postpartum the mean time for introducing PBF was 0.50 weeks (95% CI: 0, 1.02) according to the frequent short-time recalls and 1.51 weeks (95% CI: 0.66, 2.35) according to the recall since-birth (Mantel-Cox test, p = 0.049) (Figure 1). The mean time for introducing MF was 4.07 weeks (95% CI: 3.38, 4.77) according to the frequent short-time recalls and 4.50 weeks (95% CI: 3.93, 5.07) according to the recall since-birth, (Mantel-Cox-test, p = 0.82) (Figure 2).

**Figure 3:**
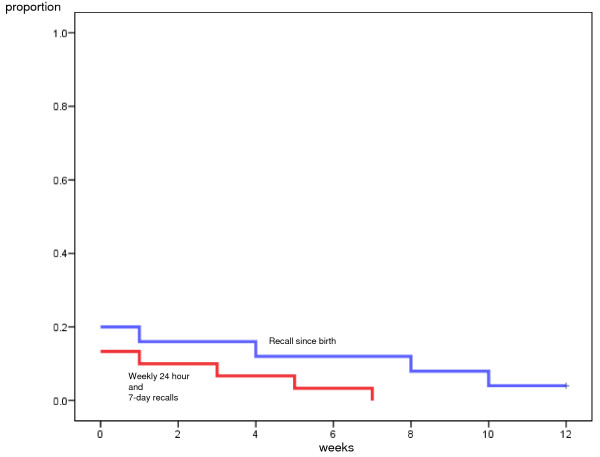
**Comparison of short-time and 'since birth' dietary assessments for introduction of predominant breastfeeding (PBF), 12 weeks**. Kaplan-Meier curves illustrating proportion introducing water, water-based drinks, oral rehydration salts or fruit juices to breastfed infants (PBF) according to the frequent short-time recall, 24-hour recall and weekly 1-week recall (red) and recall 'since-birth' (blue) dietary assessments at twelve weeks.

**Figure 4:**
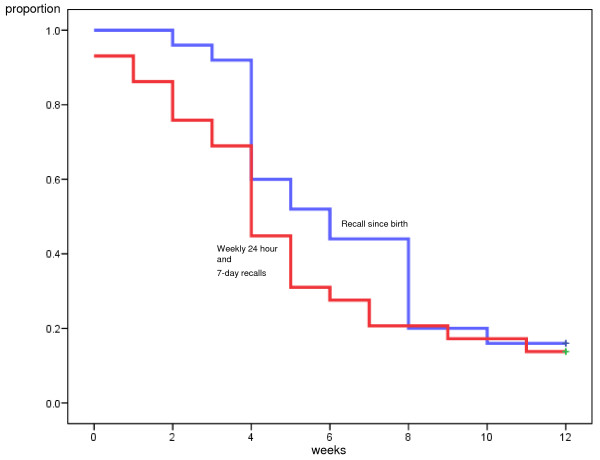
**Comparison of short-time and since birth dietary assessments for introduction of mixed feeding (MF), 12 weeks**. Kaplan-Meier curves illustrating proportion introducing milk and semi-solid feeds to breastfed infants (MF) according to the frequent short-time recall, 24-hour recall and weekly 1-week recall (red) and recall 'since-birth' (blue) dietary assessments at twelve weeks.

At twelve weeks postpartum the mean time for introducing PBF was 0.53 weeks (95% CI: 0, 1.11) according to the frequent short-time recalls and 1.40 weeks (95% CI: 0.10, 2.70) according to the recall since-birth (Mantel-Cox-test, p = 0.147) (Figure 3). The mean time for introducing MF was 5.17 weeks (95% CI: 3.86, 6.49) according to the frequent short-time recalls and 6.60 weeks (95% CI: 5.40, 7.80) according to the recall since-birth (Mantel-Cox-test, p = 0.20) (Figure 4). The minor differences observed between the analysis made at week 6 and 12 week was due to different numbers censored in the Kaplan Meier analysis.

To sum up, the Kaplan Meier analysis yielded four different figures where the introduction of PBF and MF were illustrated at both week 6 and week 12 (Figures 1, Figure 2, Figure 3, Figure 4). Within each figure the two methods used, the frequent short-time recalls and the recall since-birth were given.

The median age according to Kaplan-Meier analysis given at week 6 and 12 for introducing PBF according to both the frequent short-time recalls and recall since-birth was 0 weeks (that is, within the first week after birth). The median age for introducing water-based feeds was skewed towards the left because a large proportion had given pre-lacteal feeding and reported that information in the 1-week recall at the assessment the first week. The median age for introducing MF was 4 weeks according to the frequent short-time recalls at six and twelve weeks, and 5 and 6 weeks according to the recall since-birth at six and twelve weeks, respectively.

**Figure 5:**
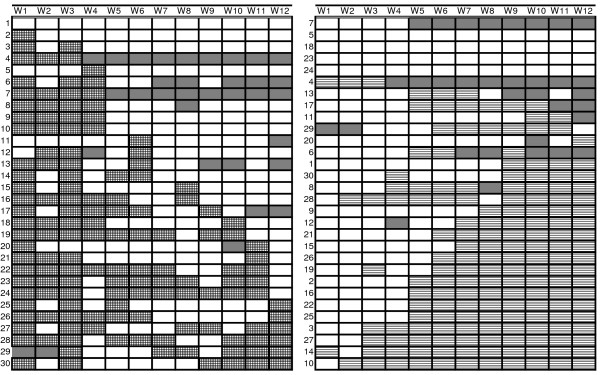
**a-b: Use of items qualifying for predominant breastfeeding (PBF) and mixed feeding (MF) for individuals by weekly assessment**. The illustrations represent water-based items, ORS and fruit juices among breastfed infants (qualifying for PBF) (grid pattern, left, 5a) and milk-based and semi-solid items among breastfed infants (qualifying for MF) (line pattern, right, 5b) given to 30 Ugandan infants from the age of 0 to 12 weeks. The information is based on weekly short-time recall (24-hour recall and 1-week recall). Each row represent one infant and each column represent one week in Figure a and b, respectively. The individuals in the two figures are linked with given numbers from 1-30. Gray pattern represents missed visits.

Whether predominant or mixed feeds were given continuously in addition to breast milk after they were introduced was illustrated by marking foods qualifying for PBF and foods qualifying for MF with different patterns. Missed interviews were also marked (Figure 5). The illustrations show a tendency for the group towards reduced usage of feeds qualifying for PBF when MF was introduced. The individual's habits were marked with numbers from 1 to 30 in the respective figures. A tendency to continue MF after its introduction was also observed.

### Specificity and sensitivity

The specificity at week 6 for being categorised as 'non-EBF' in the recall since-birth among those who were categorised as 'truly non-EBF' in the frequent short-time recall was 85.2%. The corresponding figure for 'non-MF' versus 'truly non-MF' was 90.0%. The sensitivity for being categorised as 'EBF' and 'MF' in recall since-birth were identical with the 'truly EBF' and 'truly MF' in the frequent short-time recall. This was observed both at the week 6 and the week 12 assessments.

### Reproducibility

Questions on pre-lacteal feeding and initiation of breastfeeding were asked at the 1 week visit and repeated at the 3 week visit. The results are given in Table 3. Six items not given according to the 1 week pre-lacteal feeding recall were reported the same way at the 3 week pre-lacteal feeding recall. A high proportion reported concordant answers at week 1 and 3 for diluted cow's milk and oral rehydration salts (ORS), and the answers were significant at the 0.05 level, which is at least 70% concordant. This was not the case for water or water with sugar or glucose. Significant concordant answers were seen for handling of colostrum, but not for giving pre-lacteals and timing for putting the baby to the breast. The latter two practices yielded significantly reproducible answers according to the traditional kappa test.

**Table 3:** Comparison of answers about early infant feeding practices at week 1 and 3

*Question*	*1 week*	*3 week*	*Κ, p*	*Proportion concordant, 95%CI*	Proportion test, p*
*Initiation of breastfeeding*					
When put baby to the breast after delivery			<0.01	0.75 (0.57-0.92)	0.28
Within 2 hours	17	16			
After 2 hours	11	13			
					
*Colostrum*					
Did you give the first milk to the baby or did you express and discard it?			0.03	0.89 (0.77-1.01)	0.01
Gave the first milk	26	26			
Express and discard the first milk	3	2			
					
*Pre-lacteal feeds*					
Within the first 3 days was the baby given anything to drink other than breast milk?			<0.01	0.79 (0.65-0.94)	0.14
Yes	20	20			
No	9	9			
					
Water?			<0.01	0.79 (0.65-0.94)	0.14
Yes	18	18			
No	11	11			
					
Water with sugar or glucose?			0.17	0.72 (0.56-0.87)	0.39
Yes	6	8			
No	23	21			
					
Diluted cow's milk?			NA	0.97 (0.90-1.03)	<0.01
Yes		1			
No	29	28			
					
Oral rehydration salts?			<0.01	0.96 (0.89-1.03)	<0.01
Yes	3	2			
No	26	27			
					
Not given					
Undiluted cow's milk	29	29	PA	1.0	PA
Infant formula	29	29	PA	1.0	PA
Any other powdered milk	29	29	PA	1.0	PA
Any porridge	29	29	PA	1.0	PA
Any soup	29	29	PA	1.0	PA
Any liquid as part of a ritual	29	29	PA	1.0	PA

Answers given about early infant feeding practices at week 1 and week 3 after birth, respectively. Cohen's kappa test was calculated as a measure of agreement (*Κ*, p) and a one-sample test of proportion was calculated for further accuracy (*proportion test, p*).

*One-sample test of proportion, level of agreement set to 0.7

NA = Not applicable

PA = Perfect agreement

Reproducibility of PBF and MF categories derived from the frequent short-time recall and the since-birth recall were assessed by comparing interviews conducted twice at week 6 and week 12. The kappa test and one-sample test of proportion were used to test intra-interviewee reproducibility of feeding modalities derived from answers from two different interviews. The answers were significantly concordant for seven out of eight comparisons according to the proportion test, while reproducibility according to the kappa test was significant for all eight comparisons (Table 4).

**Table 4:** Reproducibility of predominant breastfeeding and mixed feeding for since-birth and short-time recalls, respectively, at 6 and 12 weeks

*Status*	*Recall*	*Week*	*Κ, p*	*Proportion concordant, 95%CI*	Proportion test, p*
Predominant breastfeeding	Since-birth	6	PA	1.0	PA
Mixed feeding	Since-birth	6	<0.001	0.96 (0.89, 1.03)	0.001
Predominant breastfeeding	Short-time	6	PA	1.0	PA
Mixed feeding	Short-time	6	PA	1.0	PA
					
Predominant breastfeeding	Since-birth	12	PA	1.0	PA
Mixed feeding	Since-birth	12	PA	1.0	PA
Predominant breastfeeding	Short-time	12	0.0213	0.83 (0.66, 1.01)	0.109
Mixed feeding	Short-time	12	<0.001	0.94 (0.84, 1.05)	0.003

Reproducibility of estimated infant feeding categories from the since-birth and short-time recall at week 6 and week 12 after birth, respectively. Cohen's kappa test was calculated as a measure of agreement (*Κ*, p) and one-sample test of proportion was calculated for further accuracy (*proportion test, p*).

*One-sample test of proportion, level of agreement set to 0.7

NA = Not applicable

PA = Perfect agreement

## Discussion

The mean age in weeks for introducing feeds qualifying for PBF and MF were not statistically different at the 0.05-level at twelve weeks comparing frequent short-time recall to recall since-birth in the same population with Kaplan-Meier analysis. At six weeks, the mean age for introducing feeds qualifying for MF was not statistically different either, but there was a significantly different mean age for introducing feeds qualifying for PBF. A tendency towards an increased duration of EBF and PBF of around a week was seen for the recall since-birth compared to the short-time recalls. An interpretation of this could be that assessments of PBF and MF initiation by frequent short-time recalls and by recall since-birth do not yield significantly different results using Kaplan-Meier estimates at three months. Hence, a substantial number of visits could be saved while assessing EBF duration as part of programme evaluation.

This paper presents feeding practices of 30 mother-infant pairs and 10% of the mother-infant pairs were lost-to-follow up during twelve weeks of follow-up. The lack of statistically significant difference in the Kaplan-Meier analysis at twelve weeks in the durations of different feeding categories between the frequent short-time recall and recall since-birth assessments could be due to lack of power to detect a potential difference. Having said that, widespread and early initiation of MF was anticipated in this study based on earlier research in this area [16], and the sample size was not increased to detect differences that might have few clinical and practical implications. The difference observed of around one week was interpreted as minor in this respect.

Even if the design of the presented study could mimic a validation study where one method (here the recall since-birth) is compared against a so-called 'gold standard' (here frequent short-time recalls) we must expect a substantial amount of interference between the two methods. We would therefore be careful calling this a 'validation study,' but rather just a 'comparison study'. First: the 'gold standard' was not independent from what was being tested, i.e. it is possible that someone having reported on a weekly basis that they give water could remember that they had given water more easily while being asked about the same in a retrospective recall at a later point in time. But, one could also say that even if the mother could more easily remember that she had given a specific food item, it is less likely that she would remember whether she said 'yes' to that food item at week 3,4,5 or 6, etc. for the first time. For the Kaplan-Meier analysis this would imply an increased likelihood that both assessments reflected 'that the event had occurred,' but 'time-to-event' for the created PBF and MF variables would probably not be changed. Second: the 'gold standard' shared features of what was being tested. The 'gold standard' also being based on dietary recalls someone could argue it does not deserve the status of a 'gold standard.' To the latter argument one could mention that the WHO frequent short time recall as already tested to such a degree that it has got status as a non-invasive/non-tech valid method in field studies [22]. Other tools to validate the answers were not used, e.g. diaries and observation, this could have supported information about whether the recall strategies captured what they were intended to do. A Finnish study team presented a similar study to this one in a European setting where recall strategies were compared and called it 'relative validity.' High consistency between different recall strategies was found in this resource rich setting [28].

From the present study, we hypothesise that socio-economic settings including education level does not influence women's recall of infant feeding patterns. It was anticipated that introduction of feeds to an infant would have a 'mile-stone' importance for its mother, so she would be able to recall the age of introduction approximately. External circumstances could help her to remember when she started giving something, e.g. 'it happened when I had to go up-country' or 'the milk was not enough and I had to introduce other feed.' Further, if giving feeds was done from an early age and did not have any 'mile-stone' impact, we anticipate this would be reflected as well.

We observed a pattern of decreased usage of feeds qualifying for PBF with increased usage of feeds qualifying for MF. In addition, feeds were introduced sequentially: PBF was started prior to MF according to the frequent short-time recalls. After MF was introduced, most infants received milk-based and semi-solid feeds for the remainder of the period of observation. This finding could be in the favour of the 24-hour recall which is reporting proportion at different ages practising different feeding modalities. The question is not only whether the 24-hour recall is over-reporting EBF because it only captures the last 24-hours so previous practises are hidden. Longer duration recalls show lower proportions practising EBF, because it allows a longer period when more foods might be given to the infants. As Piwoz says 'the child can go in and out of feeding categories' [20], so it is possible that those who give other foods than breast milk infrequently appear as someone who practice EBF in the 24-hour recall. The question is also whether the 24-hour recall over-reports EBF because other feeds than breast milk is under-reported. For example, answering fatigue might cause this, e.g. when mothers are asked from long lists of food items they might rapidly answer 'no' to everything to avoid probing for frequency, etc. From the design of the study under-reporting cannot be assessed. Almroth and Latham did a number of studies in warm climates addressing the feeding needs for children. They used the retrospective recalls to a high degree, but explained the technique more as 'nutritional in depth interviews' than feeding recalls [29]. With this technique they presented results similar to those of this paper. Maybe the infant feeding assessment discussion should circle more around how to reduce bias and under-reporting during interviews just as much as around the instruments themselves? We observed a study fatigue with the prospective assessment and women said they were bored with the questionnaires. This should be discussed and taken into account before designing huge comprehensive follow-up studies. It could be better for the participants to do interviews seldom and of high quality than often and repetitive.

The sensitivity for detecting PBF and MF with recall since-birth was 100% compared to the frequent short-time recalls. The specificity was 85% for non-EBF and 90% for non-MF when the recall since-birth was compared to the frequent short-time recalls. The sensitivity was 100%. However, we would expect high agreement between the two strategies as the tests were not independent. Most likely the 'real-world' sensitivity and specificity would be lower than what is calculated here, the problem is that by conducting the study we interfered with both the 'memory' and maybe also with the natural 'practices' by making mothers more conscious about what they is giving.

Addressing the reproducibility of answers yielded at two different points in time was done for the variables created at 6 and 12 weeks and early feeding practices. For the variables addressing infant feeding modalities a high agreement was reached. Probably the high agreement was influenced by a relatively short time between the interviews (early morning versus late afternoon). The mother could maybe remember in the evening what she had said in the morning, this could be labelled so-called 'repetitive recall bias.' On the other hand this high degree of reproducibility is promising reflecting that mothers give consistent answers. Comparing early infant feeding practices (pre-lacteal feeding, handling of colostrum and initiation of breastfeeding) at weeks 1 and 3 did not show complete agreement for a few items. This could be interpreted as a need to catch information about early infant feeding practices as soon as possible after the first week for programs promoting and monitoring safer early infant feeding practices. Some studies have indicated that small changes in early infant feeding practices might have a huge public health impact [30]. This quantitative study only assessed 'colostrum' superficially according to the wording and categories in Table 3. However, qualitative research from the same area (unpublished) indicates that a wide variety of practices existed in the area from someone considering 'colostrum' as 'dirt' till 'squeezing' milk out to relieve the pressure. Many women, however, just treated the colostrum as 'anything coming from the breast is good.' Early infant feeding practices and data collection techniques should receive more attention in the years to come, but most of all awareness needs to be increased in low-income settings around the benefits of immediate and exclusive breastfeeding.

## Conclusion

This paper compared frequent short-time recalls with dietary recall since-birth and found overlapping patterns and a tendency towards minor increase in duration of EBF and PBF using the since-birth recall. Further studies would be needed in order to address whether the less expensive retrospective cross-sectional design following after a 24-hour dietary recall could replace prospective resource-demanding designs in population studies. This study suggests it as a useful alternative for program-evaluation purposes promoting EBF. In addition, the strategy could yield more differentiated information from cross-sectional studies and would hardly add costs. Lastly, it is perceived as more participant friendly than the frequent repetitive assessment.

## Competing interests

The authors declare that they have no competing interests.

## Authors' contributions

IE was active during the design, implementation, analysis and writing. RS contributed to the analysis and writing. AES contributed during design, interpretation, and writing. JKT and TT contributed to the intellectual content. All authors read and approved the final manuscript.
